# Plasma exchange in critically ill COVID-19 patients improved inflammation, microcirculatory clot formation, and hypotension, thereby improving clinical outcomes: fact or fiction?

**DOI:** 10.1186/s13054-020-03262-1

**Published:** 2020-09-07

**Authors:** Patrick M. Honore, Leonel Barreto Gutierrez, Luc Kugener, Sebastien Redant, Rachid Attou, Andrea Gallerani, David De Bels

**Affiliations:** ICU Department, Centre Hospitalier Universitaire Brugmann-Brugmann University Hospital, Place Van Gehuchtenplein, 4, 1020 Brussels, Belgium

We read with great interest the recent article by Morath et al. who conclude that plasma exchange (PE) improved inflammation, microcirculatory clot formation, and hypotension, thereby improving clinical outcomes [1]. We would like to make some comments. This is a good example of when misinterpretation of the results can lead to the wrong conclusions. PE has a cutoff of 1,000,000 daltons (Da) and can therefore remove many substances. Let us just take the example of the inflammatory mediators C-reactive protein (CRP) and interleukin-6 (IL-6). CRP, in its pentameric form, has a molecular weight of 120,000 Da and in its monomeric form 22,000 Da [2]. IL-6 has a molecular weight of 21,000 Da [3]. It stands to reason that these two inflammatory molecules will be easily removed by PE. Reduction of the plasma level of inflammatory mediators via the use of PE does not necessarily equate to an improvement in the septic status of the patient. It is simply an artificial reduction, “treating the numbers” so to speak. The same is true for ferritin (474,000 Da), LDH (144,000 Da), and D-dimers (180,000 Da), where the observed reduction is simply a consequence of removal and not an improvement of the patient’s condition. It is also important to note that PE has the potential to cause harm by diluting or attenuating the patient’s adaptive response to infection via depletion of immunoglobulins and complement components 3 and 4 in individuals treated with plasmapheresis [4]. Importantly, in the case of patients with COVID-19, PE will remove the protective antibodies formed by the patient, which is not desirable. Indeed, PE may not restore immune homeostasis but may rather aggravate immunoparalysis [5]. Look also at the various additional treatments received by the patients: tocilizumab, interferon, prednisolone, immunoglobulins, and convalescent serum [1]. Most of these additional treatments will be easily removed by PE. The authors stated that clinical improvements were achieved with only 1 to 2 PE, possibly indicating a direct pathophysiological influence of PE on the COVID-19-associated cytokine storm-like clinical syndrome [1]. We doubt that this is the case. The only positive effect that we can see is in the control of temperature; perhaps by inducing relative hypothermia, PE resulted in peripheral vasoconstriction responsible for the weaning of vasopressors.

## Data Availability

Not applicable.

## Abbreviations

PE: Plasma exchange
Da: Daltons
CRP: C-reactive protein
IL-6: Interleukin-6
LDH: Lactate dehydrogenase
